# Multifractal Heart Rate Value Analysis: A Novel Approach for Diabetic Neuropathy Diagnosis

**DOI:** 10.3390/healthcare12020234

**Published:** 2024-01-17

**Authors:** Andrea Coppola, Sergio Conte, Donatella Pastore, Francesca Chiereghin, Giulia Donadel

**Affiliations:** 1 Department of Systems Medicine, University of Rome Tor Vergata, 00133 Rome, Italy; andrea.coppola@inmi.it; 2 Faculty of Medicine and Surgery, Catholic University “Our Lady of Good Counsel”, 1000 Tirana, Albania; sergioconte36@gmail.com; 3 Department of Human Sciences and Quality of Life Promotion, San Raffaele University, 00166 Rome, Italy; donatella.pastore@uniroma5.it (D.P.); francescachiereghin93@gmail.com (F.C.); 4 Department of Clinical Sciences and Translational Medicine, University of Rome Tor Vergata, 00133 Rome, Italy

**Keywords:** type 2 diabetes mellitus, autonomic neuropathy, heart rate variability (HRV), Fourier analysis, fractal analysis, multifractal analysis

## Abstract

Type 2 diabetes mellitus (T2DM) is characterized by several complications, such as retinopathy, renal failure, cardiovascular disease, and diabetic neuropathy. Among these, neuropathy is the most severe complication, due to the challenging nature of its early detection. The linear Hearth Rate Variability (HRV) analysis is the most common diagnosis technique for diabetic neuropathy, and it is characterized by the determination of the sympathetic–parasympathetic balance on the peripheral nerves through a linear analysis of the tachogram obtained using photoplethysmography. We aimed to perform a multifractal analysis to identify autonomic neuropathy, which was not yet manifest and not detectable with the linear HRV analysis. We enrolled 10 healthy controls, 10 T2DM-diagnosed patients with not-full-blown neuropathy, and 10 T2DM diagnosed patients with full-blown neuropathy. The tachograms for the HRV analysis were obtained using finger photoplethysmography and a linear and/or multifractal analysis was performed. Our preliminary results showed that the linear analysis could effectively differentiate between healthy patients and T2DM patients with full-blown neuropathy; nevertheless, no differences were revealed comparing the full-blown to not-full-blown neuropathic diabetic patients. Conversely, the multifractal HRV analysis was effective for discriminating between full-blown and not-full-blown neuropathic T2DM patients. The multifractal analysis can represent a powerful strategy to determine neuropathic onset, even without clinical diagnostic evidence.

## 1. Introduction

Type 2 diabetes mellitus (T2DM) is a chronic disease characterized by high blood glucose levels that can lead to insulin resistance and consequently to low-grade inflammation and long-term complications [1]. Chronic hyperglycemia can cause long-term damage, dysfunction, and dysfunction/impairment in various organs, including the eyes, kidneys, nerves, heart, and vessels. Among long-standing T2DM associated complications, diabetic autonomic neuropathy (DAN) is the most common [2] in T2DM-diagnosed patients with poor glycemic control [3]; the pathophysiological alterations related to the onset of diabetic neuropathy are caused by a blood flow reduction on nerve capillaries that progressively leads to a fiber demyelination and axonal degeneration [4]. DAN development is tightly related to T2DM progression and glycometabolic unbalance, since almost 60% of T2DM-diagnosed patients show neuropathic symptoms [5]. Diabetic neuropathy can affect both the somatic (somatosensory diabetic neuropathy) and neurovegetative nervous system (autonomic diabetic neuropathy). Autonomic diabetic neuropathy can significantly damage the neurovascular system and all its associated systems, especially the gastrointestinal and cardiovascular system [6].

DAN is one of the main causes of sudden death associated with cardiac arrhythmias [7]. To date, DAN is neither pharmacologically treatable nor reversible, especially after a significant neuronal loss. The early diagnosis of DAN could represent a powerful tool to prevent poor T2DM prognosis. On the other hand, DAN onset has been shown to be related to age, poor glycemic control, as well as T2DM period length [8] and/or T2DM associated complications such as retinopathy or neuropathy [9,10].

Furthermore, recent evidence strongly suggests that it is crucial to adopt a comprehensive preventive approach for asymptomatic patients in order to prevent DAN progression and the subsequent requirement for surgical intervention [8]. Heart rate dysregulation is a characteristic of DAN, which is caused by an imbalance in the sympathetic/parasympathetic nervous system [11]. In this context, the Heart Rate Variability (HRV) analysis represents a cheap and powerful tool for the detection of both cardiac and autonomic diabetic neuropathy (DCAN) [12,13,14,15,16]. HRV is defined as a variation in the beat-to-beat interval, and it is usually analyzed using Fourier transform techniques to obtain the HRV spectral density power [17]. It can be analyzed both in the time and frequency domain; R wave to R wave(R–R) distance is the most important time-domain measure. The R–R parameter can be represented as a function of the heartbeat number to obtain a tachogram, which can be electronically resampled using the Fourier transform calculation. On the other hand, the Fourier transform calculation of the HRV frequency-domain analysis of the tachogram can be used to determine the spectral power parameter that represents the distribution of the frequency components of the tachogram and contains the essential information for estimating the balance between the sympathetic and parasympathetic nervous systems. The spectral power parameter is normally used in the frequency domain to express the power of the frequencies between 0.01 and 0.4 Hz.

However, since Fourier transform techniques are more suitable to resample linear signals, the analyses performed using these methods could lead to a loss of information due to the non-linearity characteristics of the tachogram.

The multifractal analysis has been proposed as a better tool to analyze these kinds of signals [18]. Recently, several papers have shown how variations in HRV can be associated with liver cirrhosis [19] and septic shock [20]. The HRV multifractal analysis has been also used to predict mortality in intensive care unit cardiovascular patients [21].

Since Ivanov and co-workers demonstrated that the tachogram is a non-linear, multifractal biomedical signal [22], we used the multifractal analysis to assess whether it is suitable for discriminating between normal patients with an unclear pathology and with overt pathology patients. Considering DAN’s serious T2DM complications and its consequences for public health, developing new strategies for the early and quick diagnosis of DAN has become mandatory [23,24]. The multifractal analysis may offer a more appropriate approach for identifying patients with DAN, compared to the linear Fourier transform analysis.

The aim of our study was to determine whether a method based on HRV could be useful to identify the progression of a disease prior to the manifestation of complete symptoms. By employing such an approach, we could effectively prevent the deterioration of the autonomic nervous system.

## 2. Materials and Methods

### 2.1. Study Population

Our study involved a total of 30 enrolled participants attending the Heart-Brain Diagnostic Center in Casarano, Lecce, Italy from 15 January to 30 April 2018.

The participants were divided into three groups. The first group consisted of 10 healthy individuals; the second group consisted of 10 patients with T2DM who had been diagnosed for 8 years but did not show any symptoms of neuropathy. These patients were considered to have a partial impact from the disease (not full-blown). The third group included 10 patients with T2DM who had been diagnosed for 9 years and exhibited at least one symptom of neuropathy, such as excessive sweating in the hands and feet, peripheral sensory motor neuropathy, or erectile dysfunction. These patients were classified as fully affected by the disease (full-blown) (https://doi.org/10.1186/s42466-020-00064-2).

All the enrolled participants gave their informed consent for our experimental protocol. T2DM-diagnosed patients (https://diabetesjournals.org/care/article/46/Supplement_1/S19/148056/2-Classification-and-Diagnosis-of-Diabetes, accessed on 12 December 2022) were treated with Metformin 1000 mg/die and Januvia (100 mg/die).

All participants enrolled in the study exhibited blood pressure values within the normal physiological range. The exclusion criteria we applied were as follows: psychological and psychiatric disorders, alcohol and tobacco use, CVD diseases, coronary heart disease, heart failure, heart arrhythmia, Q wave abnormalities, Wolff–Parkinson–White syndrome, and cardiac hypertrophy. Patients with less than 90% of the average R–R interval were also excluded.

### 2.2. Photoplethysmography Recording

To perform the photoplethysmography recording, we used the polygraph from the New Visual Energy Tester of Elemays Instruments, which was wired to a plate-embedded plethysmographic photodiode that is capable of measuring finger blood volume. The photoplethysmography recording was sampled at 128 Hz and was performed early in the morning for each participant. During the recording stages, all patients were sitting comfortably in an environment with internal ambient light and adequate internal room. Drinking or eating was not allowed up to one hour before the exam. No conversation was allowed during recording. Pulse Wave (PW) measurements, representing the pulsating peripheral blood flow, were recorded by the Plethysmogram instrument and analyzed by instrument’s software.

### 2.3. HRV Analysis

PW measurements are mandatory to estimate HRV using the R–R interval spectral analysis. The R–R intervals were automatically detected on the PW measurements using the polygraphic instrument and expressed graphically with intervals of milliseconds (ms). The standard deviation of the R–R (SDRR) was also estimated automatically. Subsequently, the HRV analysis was performed automatically by the Discrete Fourier Transform (DFT) technique to obtain the tachogram’s power spectral density (PSD) as a function of the frequency. According to the literature [25], the PSD is divided in three bands of frequency: 1—Very Low Frequency (VLF) (0.01 up to 0.04 Hz). It has been studied that thermoregulation affects VLF heart rate variability as well as Endocrine factors including thyroxine, reproductive hormones, the renin–angiotensin system, steroids, and others; 2—Low frequency (LF) (0.05 up to 0.15 Hz). This refers mainly to sympathetic system activity and baroreceptor regulation; and 3—High frequency (HF) (0.16 up to 0.4 Hz). This refers to parasympathetic system activity. Consequently, LF and HF bands represent the tachogram frequency components that allowed us to estimate sympathetic/parasympathetic balance; this was calculated using the LF/HF ratio [26,27]. We reported the results of our spectral linear analysis in healthy and pathological patients. Subsequently, we proceeded to conduct the tachogram multifractal analysis by calculating the q value and Hurst index: the q value represented the order of moments of distribution in the multifractal analysis, while the Hurst index (h) referred to the fractal dimension, which is a statistical index of system complexity. The Hurst index values range from 0 to 1. Particularly, when h values ranged from 0 to 0.5, there was a non-persistent dynamic in the tachogram referring to an increase in the R–R value, followed by a decrease or vice versa. On the other hand, when h values ranged from >0.5 to 1, there was a persistent dynamic in the tachogram, revealing an increase in the R–R value, followed by a further increase in the R–R value or vice versa. An h = 0.5 value denotes a condition called random walk, or white noise, for the given time series. It refers to a stochastic process that does not allow for the prediction of any increase or decrease in the R–R value [28]. The multifractal analysis was based on the calculation of the following parameters: h(q) index calculation, which represents the q-order Hurst exponent; τ(q) value, which represents multifractal strength; α(q), which represents the Holder exponent of referring to local singularity; and f(α), which expresses α(q) global singularity. According to multifractal theory, h(q) must be variable, depending on the q value. Moreover, the τ(q) and α(q) index must be nonlinear. f(α) must result in a concave parabola facing downwards. The multifractal analysis was performed using Δα and Δf evaluation. The multifractal calculation method was developed by Fang Wang and colleagues, [29] using Mat Lab software MFDFA (version 3.0).

### 2.4. Statistical Analysis

Data obtained with linear and multifractal HRV analysis were tested using the two-way ANOVA test and expressed as mean ± standard deviations. A *p* value < 0.05 has been considered statistically significant (Supplementary Tables S1–S3).

## 3. Results

### 3.1. Linear Analysis Is Not Able to Discriminate Asymptomatic Neuropathy Patients to Healthy Patients

In Table 1, the average values obtained from the first group (healthy participants) are reported. The HRV was 74.86 ± 6.51 beats per minute (bpm) with a tachogram standard deviation of 50.11 ms. The average total spectral power was 2540.30 ± 537.52 ms^2^/Hz; the VLF was 954.60 ± 491.60 ms^2^/Hz. For the LF and HF bands, the average values were as follows: LF = 818.00 ± 134.99 ms^2^/Hz and HF = 698.9 ± 235.12 ms^2^/Hz. Then, we normalized our results as already reported [26], and we obtained the following values: LF = 0.55 ± 0.08 ms^2^/Hz and HF = 0.45 ± 0.08 ms^2^/Hz. The sympathetic/parasympathetic balance ratio is equal to 1.28 ± 0.38. The HRV parameters obtained from the second group are displayed in Table 2: the average HRV value was 79.29 ± 10.80 bpm, and the tachogram standard deviation was equal to 47.53 ms. The average spectral power was 2225.57 ± 808.4 ms^2^/Hz, the VLF was 575.97 ± 431.96 ms^2^/Hz, the LF band was 998.90 ± 389.41 ms^2^/Hz, and the HF band was 642.70 ± 220.95 ms^2^/Hz. In normalized units, the average values were 0.60 ± 0.09 for the LF band and 0.40 ± 0.09 for the HF band. The LF/HF balance ratio was 1.76 ± 1.16. The HRV parameters obtained from the third group are displayed in Table 3. The average HRV value was 79.47 ± 10.49 bpm, while the tachogram standard deviation was 24.97 ms. The total spectral power was 649.70 ± 255.91 ms^2^/Hz; the VLF was 271.70 ± 117.64 ms^2^/Hz. The average values of the LF and HF bands were, respectively, LF = 230.50 ± 149.17 ms^2^/Hz and HF = 107.90 ± 70.34 ms^2^/Hz. In normalized units, we have the following values: LF = 0.65 ± 0.11 ms^2^/Hz and HF = 0.35 ± 0.11 ms^2^/Hz. For sympathetic/parasympathetic balance, the average value of the LF/HF obtained was 2.24 ± 1.25. The two-way ANOVA analysis did not reveal any significant variation comparing the first and the second group participants’ linear analysis (*p* = 0.546). On the other hand, the statistical analysis performed on the spectral analysis comparing the first and the third group revealed statistically significant differences (*p* < 0.0001). The statistical analysis performed on the spectral analysis comparing the second and the third group revealed statistically significant differences (*p* < 0.0001), as shown in Supplementary Tables S1–S3.

### 3.2. Multifractal Analysis Is Suitable to Distinguish Not-Full-Blown Neuropathy Patients to Full-Blown Neuropathy Patient

**Figure 1:**
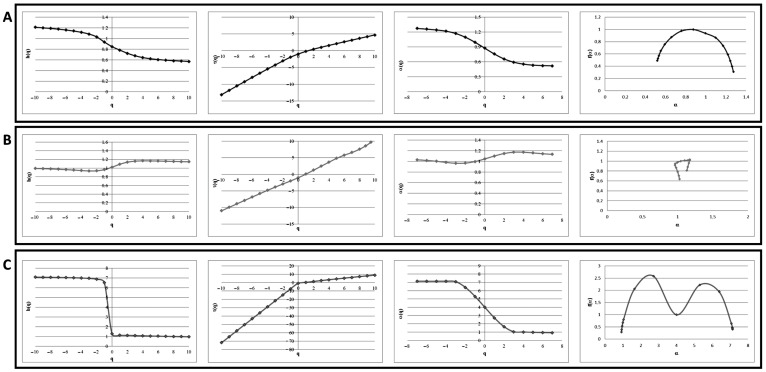
Multifractal analysis for healthy patients and diabetic patients. Panel (**A**): h(q), τ(q), α(q), and f(α) values for healthy control patients. Panel (**B**): h(q), τ(q), α(q), and f (α) values for not-full-blown diabetic patients. Panel (**C**): h(q), τ(q), α(q), and f(α) values for full-blown diabetic patients. Representative data for 10 healthy subjects (Panel (**A**)) or patients (Panels (**B**,**C**)).

Since the linear HRV Fourier analysis failed to distinguish between neuropathic T2DM full-blown and not-full-blown T2DM patients, we performed a tachogram multifractal analysis on our cohorts (Figure 1). For each healthy subject or neuropathic (full-blown, or not-full-blown) patient, we calculated variable values that characterize the multifractal analysis: h(q), τ(q), α(q), and f (α). We also reported a strong difference comparing all these parameters: healthy patients’ h(q) values showed a multifractally fashioned monotonical descending trend; conversely, their τ(q) values showed a multifractally fashioned monotonical ascending trend; and their α(q) values showed the same descending trend displayed for h(q), while the f(α) spectrum appeared as a multifractal because of the α-dependent parabolical trend. For the not-full-blown T2DM neuropathic patients, their h(q) values showed a constant trend as the q value varies, while the τ(q) and α(q) spectra were both linear. Taken together, these data showed a non-multifractal trend for f(α), since the downward concave parabolical shape was not recognizable in these tachograms. For these patients, the multifractal α(q) spectra degenerated toward a fractal shape. Then, we analyzed the full-blown T2DM neuropathic patients and we demonstrated that the h(q), τ(q), α(q), and f(α) parameters were markedly different compared to the not-full-blown neuropathic patients: their h(q) values showed a bilinear trend with a constant value of h(q) around 1, while the other linear branch of the function was assessed between 3 and 7 h(q) values. The τ(q) and α(q) values showed a bilinear trend; however, the τ(q) spectra showed a bilinear ascendant trend, while the α(q) spectra had a bilinear descendant trend. In the full-blown T2DM neuropathic patients, the f(α) parameter appeared as a double downward concave parabolic shape. Taken together, these data, shown in Figure 1, allowed us to perfectly distinguish between the not-full-blown and full-blown T2DM neuropathic patients.

## 4. Discussion

In recent decades, the analysis of heart rate variability (HRV) has emerged as a potent technique for assessing the functioning of the autonomic nervous system. It enables the identification of dysregulation in the balance between the sympathetic and parasympathetic branches of the nervous system [30,31]. Lately, HRV has been widely used in the diagnosis of T2DM-related complications, such as autonomic diabetic neuropathy, since it offers exceptional ease of use and remarkable sensitivity, compared to other methods [14]. Several analysis methods have been proposed for HRV; we used the linear and multifractal method. Our preliminary data showed that the linear HRV analysis has successfully identified significant differences between healthy patients and T2DM patients with DAN symptoms. However, the linear HRV analysis method failed to discriminate between T2DM patients with full-blown DAN symptoms and T2DM patients without DAN symptoms. DAN is a serious challenge to face nowadays; the mortality rate among DAN-diagnosed diabetes patients over a period of 2.5 years has been evaluated at 27.5%. However, the overall survival rate after 5 years increased to 53% in comparison to 15% for diabetic patients with physiological autonomic functions [32]. Another study has highlighted that survival rate over a period of 5 years for asymptomatic DAN patients at 27%; interestingly, there has been no difference demonstrated in the duration of diabetes between individuals with or without DAN, as observed in deceased patients [33]. Taking these data into account, our HRV analysis technique has the potential to significantly decrease such percentages. This is because it enables the physician to easily identify the multifractal changes that indicate variations in the clinical situation. The multifractal path that characterizes asymptomatic patients can degenerate toward a recognizable path that is associated with full-blown DAN, allowing the physician to set up a new therapy that is able either to reduce or avoid DAN onset. We enrolled healthy patients around 35 years old, without any overt pathology, in order to perform a plethysmography analysis as a control, to be compared to the multifractal HRV of diabetic patients with and/or without neuropathy. Our findings show that the use of the multifractal HRV analysis successfully differentiated asymptomatic patients in our cohort prior to DAN development. According to the data obtained from the present study, the average onset of asymptomatic DAN patients is 7.9 years, whereas the average onset for symptomatic DAN patients is 9 years. Our findings suggest that the clinical condition could potentially deteriorate within approximately one year. Taken together, our data highlighted the importance of early DAN detection, emphasizing the need for a comprehensive treatment approach that effectively prevents the progression of DAN. The multifractal analysis is an extremely simple and easy diagnosis system that can provide significant indications to prevent the development of DAN. By employing this method, it becomes possible to differentiate between an asymptomatic patient and a symptomatic DAN patient without the need for any additional surveys. Future research related to this study involves forecasting an increase in the number of patients participating in population stratification for BMI, HbAC1, and various glycometabolic parameters. We are aware of the observational nature of this study, and our efforts will be focused on identifying novel and easily evaluable biomarkers, in order to establish a correlation between HRV measurement and changes in biochemical molecules.

## 5. Conclusions

Our preliminary data demonstrated that the multifractal HRV analysis, rather than the linear HRV analysis, could detect the early asymptomatic progression of DAN patients with T2DM.

## Figures and Tables

**Table 1:** Results of HRV and spectral analysis (Power Tot, VLF, LF, HF) and LF/HF ratio for 10 control patients. (u.n. = unity of normalization).

ID	Age	Sex	Heart Rate (p/m)	Tachogram Standard Deviation (ms)	Total PSD (ms^2^/Hz)	VLF (ms^2^/Hz)	LF (Symph) (ms^2^/Hz)	HF (Parasymph) (ms^2^/Hz)	LF (u. n.)	HF (u. n.)	LF/HF
1	49	M	85.3	45.8	2097	388	898	811	0.53	0.47	1.11
2	27	F	73.6	52.6	2768	838	999	875	0.53	0.47	1.14
3	32	F	68.4	54.2	2937	1251	935	645	0.59	0.41	1.45
4	43	M	73.9	45.3	2056	835	653	519	0.56	0.44	1.26
5	29	F	72.7	58.9	3478	1659	625	1118	0.36	0.64	0.56
6	20	M	66.9	51.9	2700	1518	732	402	0.65	0.35	1.82
7	31	F	83.5	49.7	2473	395	999	939	0.52	0.48	1.06
8	37	M	71.6	43.2	1874	501	817	478	0.63	0.37	1.71
9	39	F	70.4	55.3	3060	1513	758	722	0.51	0.49	1.05
10	44	M	82.3	44.2	1960	648	764	480	0.61	0.39	1.59
Average	35.10		74.86	50.11	2540.30	954.60	818.00	698.90	0.55	0.45	1.28
St. Dev.	8.89		6.51		537.52	491.61	134.99	235.12	0.08	0.08	0.38

**Table 2:** Results of HRV and spectral analysis (Power Tot, VLF, LF, HF) and LF/HF ratio for 10 T2MD diagnosed with not-full-blown autonomic neuropathy group (u.n. = unity of normalization).

ID	Age	Sex	Heart Rate (p/m)	Tachogram Standard Deviation (ms)	Total PSD (ms^2^/Hz)	VLF (ms^2^/Hz)	LF (Symph) (ms^2^/Hz)	HF (Parasymph) (ms^2^/Hz)	LF (u. n.)	HF (u. n.)	LF/HF
1	44	M	90.3	42.7	1833.00	356.00	832	645	0.56	0.44	1.29
2	40	F	87.5	43.2	1869.00	158.00	900	811	0.53	0.47	1.11
3	47	M	83.5	40.9	1686.80	123.80	832	731	0.53	0.47	1.14
4	39	F	73.6	42.5	1816.25	387.25	800	629	0.56	0.44	1.27
5	49	M	88.5	44.0	1957.23	437.23	830	690	0.55	0.45	1.20
6	35	F	68.3	65.3	4276.00	1385.00	1805	1086	0.62	0.38	1.66
7	52	M	94.4	44.1	1948.00	571.00	816	561	0.59	0.41	1.45
8	46	F	74.6	52.1	2714.41	1301.41	797	616	0.56	0.44	1.29
9	47	M	68.0	50.8	2581.00	590.00	1655	336	0.83	0.17	4.93
10	36	F	64.2	39.6	1574.00	450.00	722	322	0.69	0.31	2.24
Average	43.50		79.29	46.52	2225.57	575.97	998.90	642.70	0.60	0.40	1.76
St. Dev.	5.72		10.80		808.44	431.96	389.41	220.95	0.09	0.09	1.16

**Table 3:** Results of HRV and spectral analysis (Power Tot, VLF, LF, HF) and LF/HF ratio for 10 T2D diagnosed with full-blown autonomic neuropathy group (u.n. = unity of normalization).

ID	Age	Sex	Heart Rate (p/m)	Tachogram Standard Deviation (ms)	Total PSD (ms^2^/Hz)	VLF (ms^2^/Hz)	LF (Symph) (ms^2^/Hz)	HF (Parasymph) (ms^2^/Hz)	LF (u. n.)	HF (u. n.)	LF/HF
1	55	M	70.5	19.3	373	246	56	39	0.59	0.41	1.44
2.	50	F	72.3	24.8	617	306	157	98	0.62	0.38	1.60
3.	44	M	77.9	21.0	445	62	279	75	0.79	0.21	3.72
4	40	F	68.6	32.2	1040	360	364	280	0.57	0.43	1.30
5	52	M	100.0	30.3	922	326	390	147	0.73	0.27	2.65
6	41	F	79.6	24.0	578	193	289	62	0.82	0.18	4.66
7	43	M	88.2	30.9	959	296	476	148	0.76	0.24	3.22
8	52	F	72.0	24.8	618	373	113	95	0.54	0.46	1.19
9	54	M	91.8	16.5	274	118	58	55	0.51	0.49	1.05
10	46	F	73.8	25.9	671	437	123	80	0.61	0.39	1.54
Average	47.70		79.47	24.97	649.70	271.70	230.50	107.90	0.65	0.35	2.24
St. Dev.	5.56		10.49		255.91	117.64	149.17	70.34	0.11	0.11	1.25

## Data Availability

Data are contained within the article and Supplementary Materials.

## Supplementary Materials

The following supporting information can be downloaded at: https://www.mdpi.com/article/10.3390/healthcare12020234/s1, Figure S1: Formatting of Mathematical Components and Supplementary Tables S1–S3.
