# Supplementary material for: Multifractal Heart Rate Value Analysis: A Novel Approach for Diabetic Neuropathy Diagnosis
Source: Healthcare (Basel). 2024 Jan 17;12(2):234. doi: 10.3390/healthcare12020234 (PMC10815481; doi:10.3390/healthcare12020234)
Supplement: Supplementary file 1 [file healthcare-12-00234-s001.zip › Supplementary Tables.pdf]

Table S1. Two-way ANOVA statistical analysis. Comparison of spectral analysis (Power Tot, VLF, LF, HF) and LF/HF ratio between control subjects and T2DM diagnosed subjects with not-full blown DAN.

Two-way ANOVA	**Control subjects and T2DM diagnosed subjects with not-full blown DAN**			
Source of Variation	% of total variation	P value		
Interaction	3.92	0.94		
Spectral analysis	0.42	p=0.5465		
Participants	4.85	0.8874		
Source of Variation	P Value summary	Significant		
Interaction	Ns	No		
Spectral analysis	Ns	No		
Participants	Ns	No		
Source of Variation	Df	Sum-of-squares	Mean square	F
Interaction	9	3035000	337300	0.3832
Spectral analysis	1	322800	322800	0.3668
Participants	9	3761000	417900	0.4748
Residual	80	74010000	880100	
Number of missing values	0			

Table S2 Two-way ANOVA statistical analysis. Comparison of spectral analysis (Power Tot, VLF, LF, HF) and LF/HF ratio between control subjects and T2DM diagnosed subjects with full-blown DAN.

Two-way ANOVA	**Control subjects and T2DM diagnosed subjects with full-blown DAN**			
Source of Variation	% of total variation	P value		
Interaction	2.38	0.9739		
Spectral analysis	24.32	p<0.0001		
Participants	1.97	0.9864		
Source of Variation	P Value summary	Significant		
Interaction	Ns	No		
Spectral analysis	***	Yes		
Participants	No	No		
Source of Variation	Df	Sum-of-squares	Mean square	F
Interaction	9	1375000	152800	10.2962
Spectral analysis	1	14070000	14070000	27.28
Participants	9	1139000	1139000	0.2453
Residual	80	41260000	41260000	
Number of missing values	0			

Table S3. Two-way ANOVA statistical analysis. Comparison of spectral analysis (Power Tot, VLF, LF, HF) and LF/HF ratio between T2DM diagnose subjects with not-full blown DAN and T2DM diagnosed subjects with full-blown DAN.

Two-way ANOVA	**T2DM diagnose subjects with not-full blown DAN and T2DM diagnosed subjects with full-blown DAN**			
Source of Variation	% of total variation	P value		
Interaction	5.81	0.6684		
Spectral analysis	20.13	p<0.0001		
Participants	4.56	0.8075		
Source of Variation	P Value summary	Significant		
Interaction	Ns	No		
Spectral analysis	***	Yes		
Participants	Ns	No		
Source of Variation	Df	Sum-of-squares	Mean square	F
Interaction	9	2924000	324900	0.7431
Spectral analysis	1	10130000	1013000	23.17
Participants	9	2293000	254800	0.52828
Residual	80	34970000	437200	
Number of missing values	0			

In relation to the statistical analysis, our findings show that there was no difference in HRV (heart rate variability) between individuals in the control group and the individuals in the not full-blown group, as shown in Table S1. However, we observed a remarkably different condition in full-blown subjects, as shown in Table S2, where we observed that PSD (Power Spectral Density) resulted lower compared to both control subjects and not full-blown subjects. The LF/HF ratio revealed a significant imbalance in the sympathetic nervous system modulating component in comparison to the parasympathetic component, specifically due to an increase in the LF component with a decrease in HF. Therefore, the values of spectral distribution in full-blown subjects differed significantly from those in control subjects, as well as from the values measured in not full-blown subjects. The statistical analyses presented in Tables S2 and S3 underscores a highly significant distinction between control group and full-blown group, as well as between not full-blown group and full-blown group (Two-way ANOVA index with a value of P<0.0001).
