# Supplementary figures and images for: Multifractal Heart Rate Value Analysis: A Novel Approach for Diabetic Neuropathy Diagnosis
Source: Healthcare (Basel). 2024 Jan 17;12(2):234. doi: 10.3390/healthcare12020234 (PMC10815481; doi:10.3390/healthcare12020234)

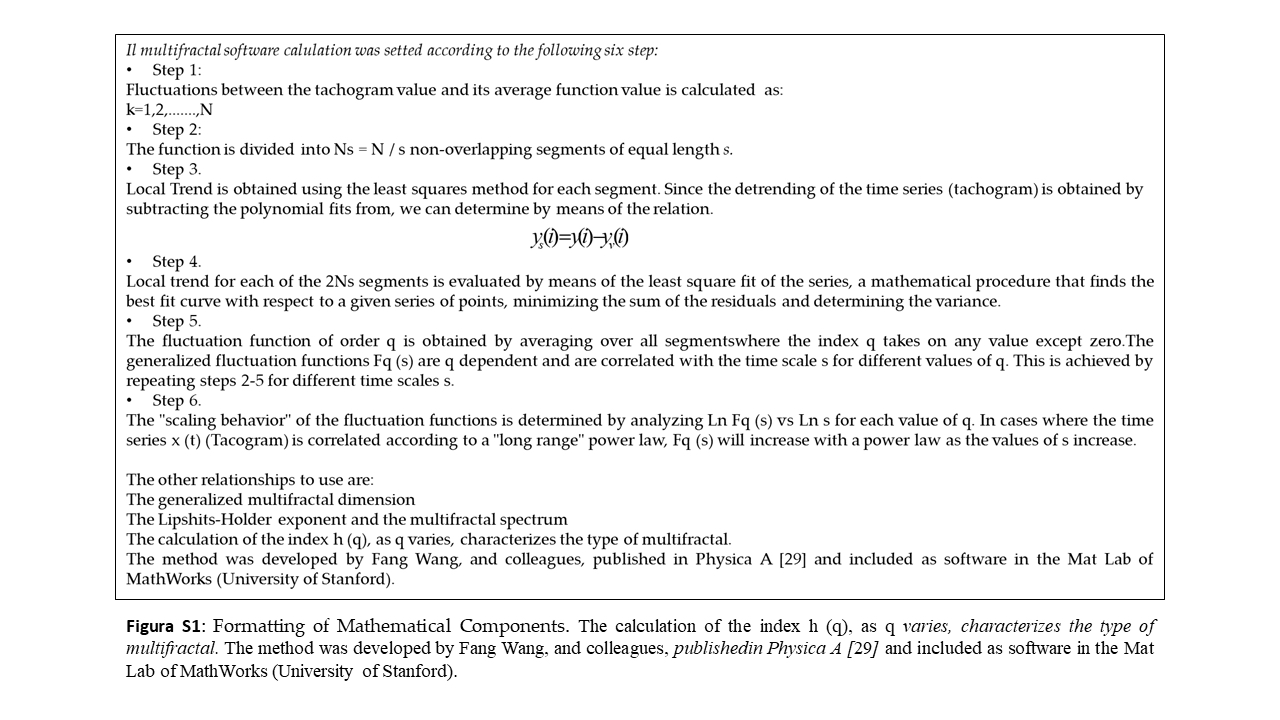

Supplement: Supplementary file 1 [file healthcare-12-00234-s001.zip › Figure S1.jpg]
